# The nanoscale organization of reticulon 4 shapes local endoplasmic reticulum structure in situ

**DOI:** 10.1083/jcb.202301112

**Published:** 2023-07-26

**Authors:** Lukas A. Fuentes, Zach Marin, Jonathan Tyson, David Baddeley, Joerg Bewersdorf

**Affiliations:** 1 Department of Cell Biology, Yale University School of Medicine, New Haven, CT, USA; 2 Department of Biomedical Engineering, Yale University, New Haven, CT, USA; 3 Auckland Bioengineering Institute, University of Auckland, Auckland, New Zealand; 4 Department of Chemistry, Yale University, New Haven, CT, USA; 5 Department of Physics, Yale University, New Haven, CT, USA

## Abstract

The endoplasmic reticulum’s (ER’s) structure is directly linked to the many functions of the ER, but its formation is not fully understood. We investigate how the ER–membrane curving protein reticulon 4 (Rtn4) localizes to and organizes in the membrane and how that affects the local ER structure. We show a strong correlation between the local Rtn4 density and the local ER membrane curvature. Our data further reveal that the typical ER tubule possesses an elliptical cross-section with Rtn4 enriched at either end of the major axis. Rtn4 oligomers are linear shaped, contain about five copies of the protein, and preferentially orient parallel to the tubule axis. Our observations support a mechanism in which oligomerization leads to an increase of the local Rtn4 concentration with each molecule, increasing membrane curvature through a hairpin wedging mechanism. This quantitative analysis of Rtn4 and its effects on the ER membrane result in a new model of tubule shape as it relates to Rtn4.

## Teaser

Fuentes and Marin et al. show that Rtn4 forms linear-shaped oligomers containing an average of five Rtn4 proteins that localize to the sides of elliptical tubules with orientations parallel to the tubule axis. These oligomers increase local curvature in the ER membrane by increasing local Rtn4 density.

## Introduction

The endoplasmic reticulum (ER) is the site of several critical cellular functions. These include the morphological regulation of other organelles by ER tubules (Abrisch et al., 2020; Friedman et al., 2011; Hoyer et al., 2018; Zheng et al., 2022), protein translation on rough ER sheets (Jan et al., 2014; Shibata et al., 2010; West et al., 2011), and lipid biogenesis in the tubule-rich ER–Golgi intermediate compartment (Glick and Nakano, 2009). The ER morphology adapts to the needs of different cell types, displaying expansive sheets for enhanced protein expression and secretion or highly tubular ER in the case of androgen production (Lee et al., 2005; Zirkin and Papadopoulos, 2018). This critical link between the ER structure and function has been made clear as disrupting the specialized processes of the ER in these cells consistently leads to perturbed ER structure (Lee et al., 2005; Zirkin and Papadopoulos, 2018). Recently, ER structure has been strongly linked to disease markers for obesity and diabetes in liver tissues by the remarkable discovery that these markers can be rescued to levels of non-diseased tissues by manipulation of ER structure alone (Parlakgül et al., 2022).

Investigations of ER morphology are, however, complicated by the fact that the sizes of typical ER features are below the diffraction limit of standard light microscopes. Studies making use of super-resolution microscopy (Nixon-Abell et al., 2016; Schroeder et al., 2019; Wang et al., 2022) or electron microscopy (Puhka et al., 2012; Terasaki et al., 2013; Zamponi et al., 2022) methods have highlighted the fact that the ER structure is more complex than once thought, as structural details of the ER were not fully appreciated when visualized with conventional light microscopy methods. For example, what were once thought to be continuous ER sheets have been revealed to often be intricate tubular matrices (Nixon-Abell et al., 2016) and sheets containing dynamic nanoholes (Schroeder et al., 2019).

The reticulon (Rtn) and REEPs/DP1/Yop1p protein families are known to be responsible for stabilizing curvature in the ER membrane (Voeltz et al., 2006; Hu et al., 2008). Both protein families possess reticulon homology domains (RHD) with hairpin topologies that do not completely pass through the membrane bilayer (Voeltz et al., 2006; Zurek et al., 2011). This results in the proteins forming wedges in the ER membrane that displace more phospholipids in the cytosolic leaflet than the luminal leaflet. This wedging mechanism is likely a major contributor to the membrane-curving function of these proteins. REEPs/DP1/Yop1p have been shown to form dimers that may organize into a splayed conformation to promote membrane curvature (Wang et al., 2021). However, reticulons and REEPs/DP1/Yop1p have also been shown to form higher-order homo-oligomers in vitro, and it was proposed that these oligomers form arches in the membrane to scaffold it into a curved topology (Shibata et al., 2008; Hu et al., 2008). Due to the difficulty of visualizing such structures at the size scale of tens of nanometers, neither model has been confirmed yet by direct visualization of these complexes.

Reticulon 4 (Rtn4) is one of the best-studied proteins among the reticulon and REEPs/DP1/Yop1p families of proteins. In addition to its well-known partitioning to tubules and sheet edges (Shibata et al., 2010; Voeltz et al., 2006), Rtn4 generally localizes to areas of higher membrane curvature (Kiseleva et al., 2007) and nanoholes within ER sheets (Schroeder et al., 2019). In wider tubules, it also displays two parallel lines along their sides (Wang et al., 2022). Rtn4’s several isoforms all possess a C-terminal RHD (Oertle and Schwab, 2003; Yang and Strittmatter, 2007; Schroeder et al., 2019) that is important for its localization as well as Rtn4’s ability to curve membranes, which suggests all RHD-containing proteins share a general mechanism for shaping membranes (Zurek et al., 2011), though the exact mechanism remains unclear.

In this study, we set out to understand how Rtn4 proteins are organized at the nanoscale in situ to enable their membrane-curving function and how that organization affects the local ER structure. We make use of a suite of recently developed software tools (Barentine et al., 2018; Thevathasan et al., 2019; Marin et al., 2021, 2023) and super-resolution methods (Zhang et al., 2020; Chung et al., 2022) to accomplish this. Our results lead us to propose a refined model for Rtn4 localization, organization, and its effect on tubule shape.

## Results and discussion

### Rtn4 density determines local ER membrane curvature

**Figure S1.**
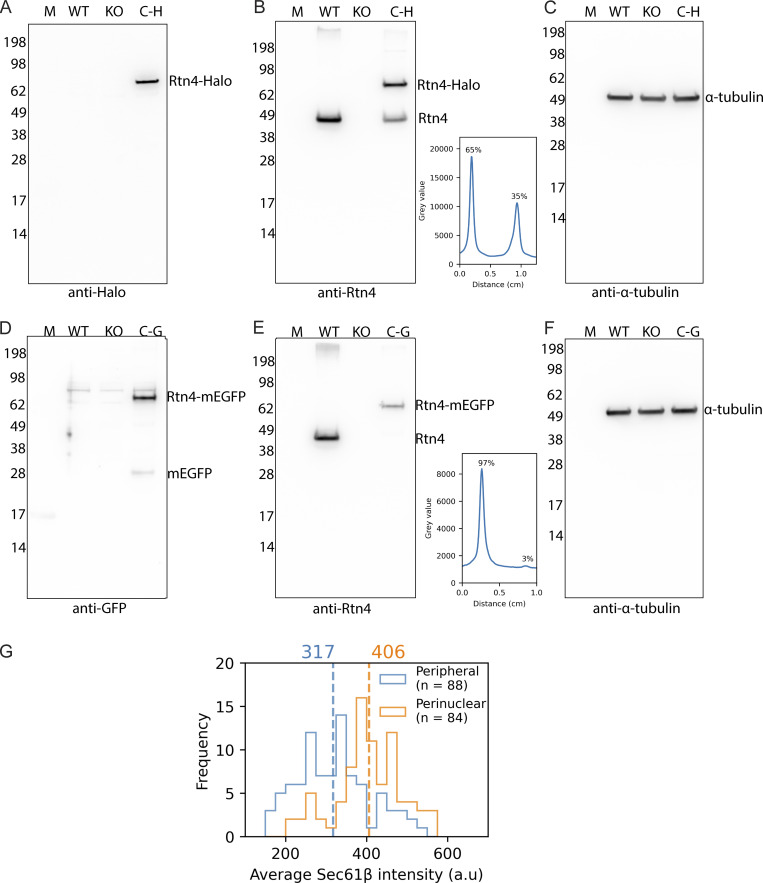
**Western blots of CRISPR cell lines. (A–C)** Probing U-2 OS Rtn4-Halo and control cell lines with anti-Halo, anti-Rtn4, and anti-α-tubulin antibodies, respectively. **(D–F)** Probing U-2 OS Rtn4-mEGFP and control cell lines with anti-GFP, anti-Rtn4, and anti-α-tubulin antibodies, respectively. **(B and E)** Line plots drawn through the U-2 OS Rtn4-Halo and Rtn4-mEGFP bands of tagged (top band/left peak) and untagged Rtn4 (bottom band/right peak), respectively. **(G)** Histogram of average Sec61β pixel intensity measured along tubules. M: Marker; WT: U-2 OS cells; KO: U-2 OS Rtn4 knock-out cells; C-H: Rtn4-Halo CRISPR clone cells; C-G: Rtn4-mEGFP CRISPR clone cells. Source data are available for this figure: SourceData FS1.

**Figure 1.**
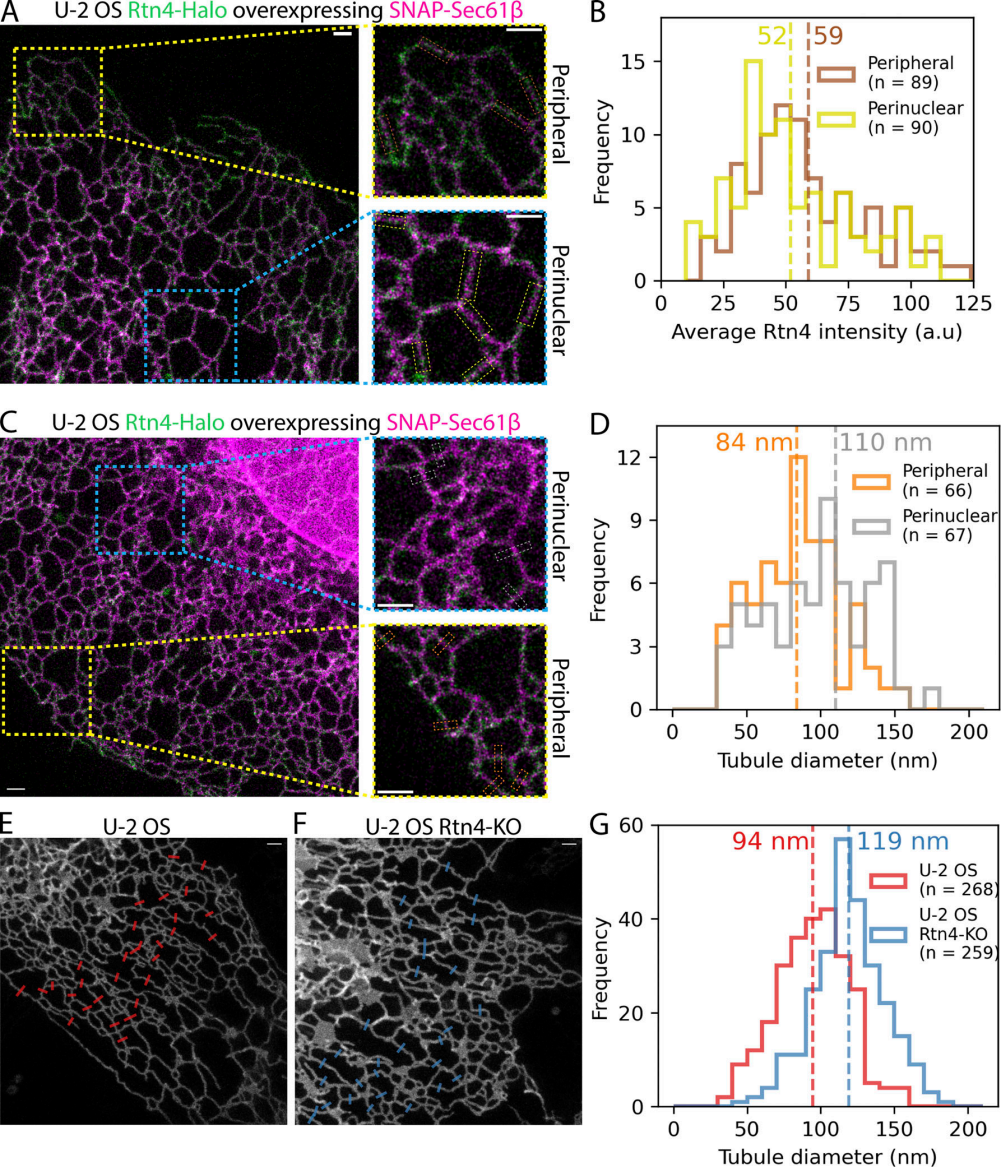
**Effects of Rtn4’s distribution on microscale ER structure. (A and C)** Two-color live-cell STED images of U-2 OS Rtn4-Halo (green) CRISPR cells overexpressing SNAP-Sec61β (magenta). **(A)** Brown and yellow boxes are highlighting where regions of interest were made for Rtn4 pixel intensity measurements in peripheral and perinuclear tubules, respectively. **(B)** Histogram of average Rtn4 pixel intensity measured along tubules (P < 0.05; Wilcoxon rank-sum statistic). **(C)** Orange and gray boxes are highlighting where line plots were made for tubule diameter measurements using the NEP fitting method based on SNAP-Sec61β fluorescence for peripheral and perinuclear tubules, respectively. **(D)** Histogram of diameters of peripheral and perinuclear tubules (P < 0.01; Wilcoxon rank-sum statistic). **(E and F)** STED images of Halo-Sec61β overexpressed in U-2 OS and U-2 OS Rtn4-KO cells, respectively. Red and blue boxes represent where line plots were created for tubule diameter measurements for each dataset. **(G)** Diameters of U-2 OS and U-2 OS Rtn4-KO cells’ tubules (P < 0.001; Wilcoxon rank-sum statistic). **(B, D, and G)** Hashed vertical lines represent the average values that appear near each line. **(A, C, E, and F)** The scale bars represent 1 µm.

We first set out to understand how Rtn4’s distribution and local abundance can affect ER structure. Using live-cell stimulated emission depletion (STED) microscopy, we imaged Rtn4 endogenously tagged with HaloTag at its C-terminus, which is shared by all Rtn4 isoforms, via CRISPR Cas9 gene editing (Fig. S1, A–C). We additionally overexpressed Sec61β, an ER membrane protein, tagged with SNAP-tag to provide information about the underlying ER structure. Analysis of the difference in Rtn4 pixel intensities along tubules revealed that Rtn4 is significantly more abundant in tubules at the outer periphery of the cell than in those that are closer to the nucleus (Fig. 1, A and B), whereas a similar analysis of Sec61β pixel intensities in U-2 OS cells overexpressing Sec61β showed the opposite distribution (Fig. S1 G). We asked if this discrepancy in the abundance of Rtn4 in different regions of the ER is correlated with differences in the underlying structures. Specifically, we hypothesized that more Rtn4 present in a tubule leads to a smaller tubule diameter. From our live-cell STED images, we measured the diameters of peripheral ER tubules and compared them with the diameters of perinuclear tubules using our previously developed NEP-Fitting tool (Barentine et al., 2018). The peripheral (Rtn4-enriched) tubules displayed significantly smaller diameters on average (Fig. 1, C and D), consistent with our hypothesis and the observed distribution of Sec61β (Fig. S1 G). However, to ensure that this difference in tubule diameters was due to varying amounts of Rtn4 alone, we imaged overexpressed Sec61β tagged with HaloTag in both live U-2 OS and live U-2 OS Rtn4 knock-out cells (Schroeder et al., 2019) and compared the diameters of their ER tubules (Fig. 1, E–G). Our analysis showed a significant difference between them with U-2 OS and U-2 OS Rtn4 knock-out cells possessing 94- and 119-nm diameter tubules on average, respectively. Emerging studies suggest that Rtn4’s effect on tubule size has a direct impact on the ER’s ability to deliver Ca^2+^ and thus axonogenesis (Konno et al., 2021 *Preprint*). Altogether, these results strongly suggest that higher Rtn4 abundance leads to thinner tubule diameters, consistent with earlier findings of in vitro studies (Hu et al., 2008).

**Figure 2.**
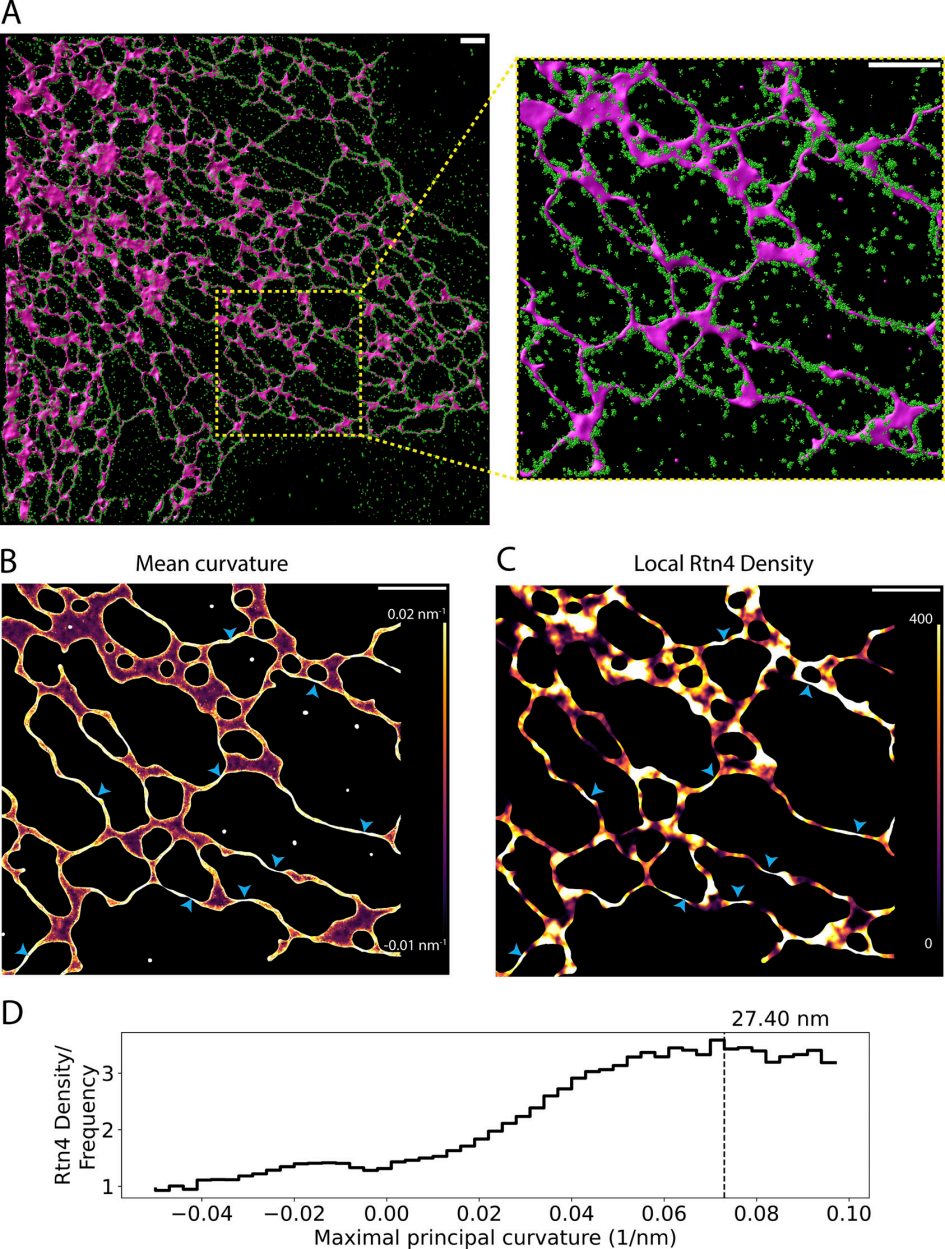
**Correlation between Rtn4’s local density and ER membrane curvature intensity. (A)** 3D data of Rtn4 localizations (green spheres) and a 3D surface based on Sec61β localizations (magenta-shaded surface). **(B)** 3D surface colored by mean curvature which ranges from −0.01 to 0.02 nm^−1^. **(C)** 3D surface colored by the local Rtn4 density within 50 nm of each vertex on the 3D surface ranging from 0 to 400 localizations. The blue arrowheads highlight locations on the surface that possess a high Rtn4 density nearby. **(D)** Histogram of the frequency of Rtn4 localizations occurring within 50 nm of a given vertex after normalizing for the frequency of the vertices' maximal principal curvatures (the curvature of the circular cross-section of the tubule). The hashed line represents the curvature where the Rtn4 density is the highest. The value above the hashed line represents the diameter of a hypothetical tubule with such a curvature. The scale bars represent 1 μm.

**Figure S2.**
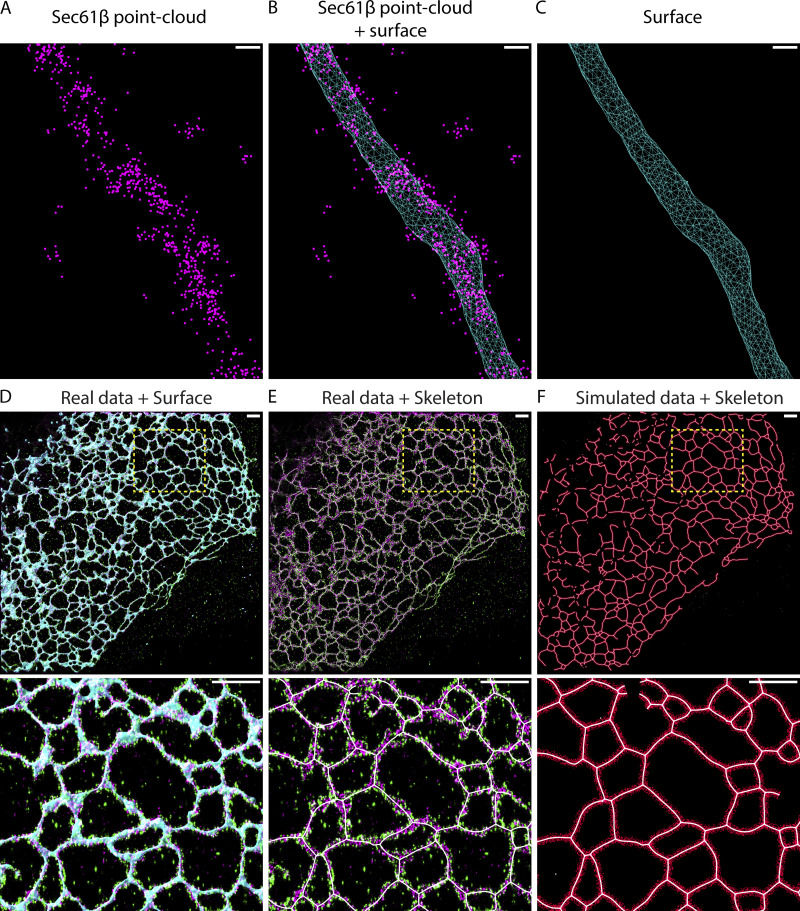
**Point cloud surface fitting, skeletons, and φ simulations. (A)** Example of a point cloud of Sec61β. **(B)** Point cloud in A and a surface generated from that point cloud. **(C)** The surface alone. The surface is composed of triangles, which are joined by shared edges. We refer to the points where multiple edges meet as vertices and use their locations to calculate the local density of Rtn4. **(A–C)** Scale bars represent 50 nm. **(D)** Two-color 3D SMLM data of Rtn4 (green), Sec61β (magenta), and a 3D surface based on the Sec61β point cloud (cyan). **(E)** As in D but with the 3D surface removed and the skeleton (white) added. **(F)** Skeleton (white) is shown with data simulated randomly in circular rings (red) around it. **(D–F)** Scale bars represent 1 μm.

We next investigated how the density of Rtn4 affects ER nanostructure locally by imaging endogenous Rtn4 and overexpressed mCherry-Sec61β in U-2 OS cells using 3D single-molecule localization microscopy (3D-SMLM; Huang et al., 2016; Zhang et al., 2020). We generated accurate 3D surfaces (Marin et al., 2023) based on the Sec61β point cloud to serve as a proxy for the ER membrane (Fig. 2 A and Video 1) and calculated the mean surface curvature (Fig. 2 B) and Rtn4 density within 50 nm of each vertex on the surface (Fig. 2 C and Fig. S2). Locations of high Rtn4 density consistently correlate with locations of high mean curvature (Fig. 2, B and C; blue arrowheads). To support this observation quantitatively, we examined Rtn4 density as a function of the local curvature on the 3D surface (the maximal principal curvature at each vertex on a tubule). We normalized the Rtn4 density by the frequency of the maximal principal curvatures on nearby surface vertices. This analysis revealed that as the maximal principal curvature increases, so does the Rtn4 density (Fig. 2 D). The Rtn4 density peaks at a curvature of 0.073 nm^−1^, which translates to a tubule diameter of ∼27 nm. These results suggest that the local density of Rtn4 has a significant role in determining the local membrane curvature.

**Video 1.** **Rtn4 localizations on a 3D surface.** U-2 OS cells transiently transfected with mCherry-Sec61β imaged with 3D-SMLM on our 4Pi-SMLM microscope. The dataset is also shown in Fig. 2. Rtn4 localizations (green spheres) are shown on the 3D surface based on the mCherry-Sec61β point cloud (shaded magenta surface). Playback speed is 10 frames per second.

### ER tubules have elliptical cross-sections, and Rtn4 localizes to their sides

**Figure 3.**
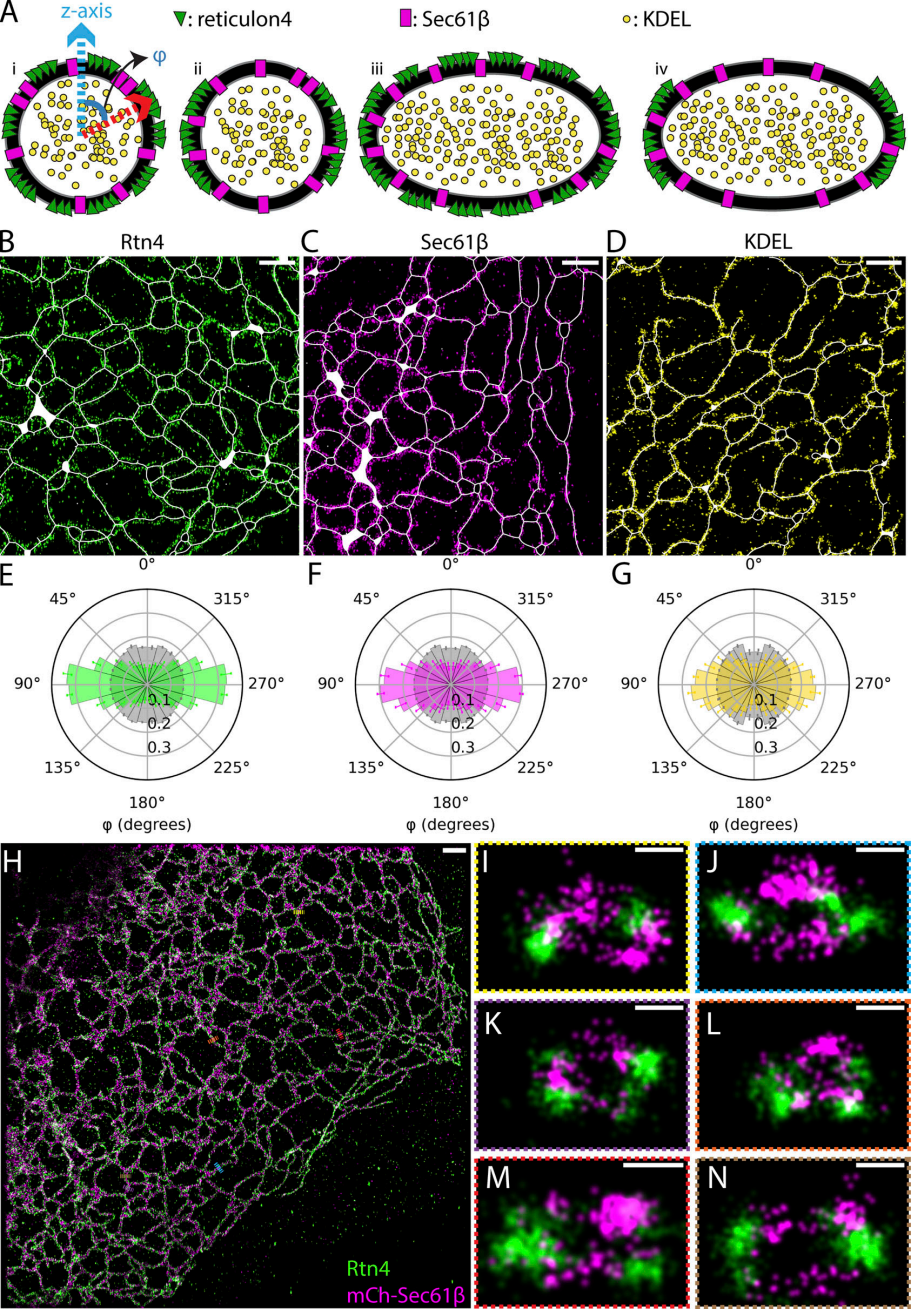
**Angular distributions of ER proteins around tubule cross-sections. (A)** Possible models of ER protein distributions in tubule cross-sections. Model (i) shows the vectors that angle φ is being calculated between. **(B–D)** Representative regions of interest taken from datasets of each protein. Skeletons, Rtn4, Sec61β, and KDEL are displayed in white, green, magenta, and yellow, respectively. **(E–G)** Polar histograms of φ. The Rtn4, Sec61β, and KDEL observed data is shown by the green, magenta, and yellow histograms, respectively. The gray histograms are from simulated datasets of points randomly distributed in a circular ring around the skeleton that was created based on each set of observed data (P <<< 0.001 for each protein’s distribution compared to their simulated random distributions; Wilcoxon rank-sum statistic; *n* = 3 cells from unique sample preparations). The Rtn4, Sec61β, and KDEL data represents 2,589,190; 1,881,961; and 2,222,140 unique angle calculations total across the *n* = 3 biological replicates, respectively. Error bars represent the standard deviation of the frequency of each histogram bin between biological replicates. **(H)** Overview of a U-2 OS cell overexpressing mCherry-Sec61β (magenta) labeled with an anti-RFP nanobody and endogenous Rtn4 (green) labeled with primary and secondary antibodies. **(I–N)** Cross-sections of ER tubules with 3D views orthogonal to the tubule axis. Data was recorded with a 4Pi-SMS microscope. Scale bar: 1 μm (B–D and H); 50 nm (I–N).

**Figure S3.**
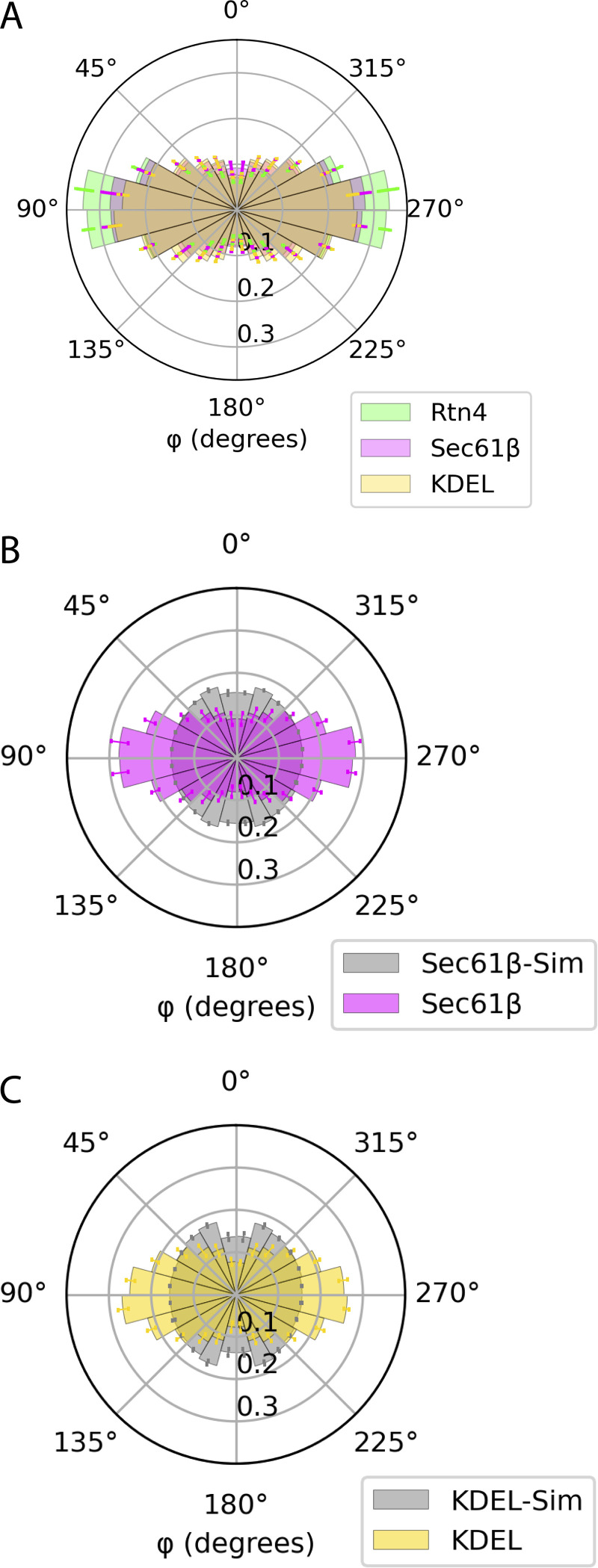
**Control histograms of φ distributions. (A)** The histograms of Rtn4 (green), Sec61β (magenta), and KDEL (yellow) together. The distributions were tested for significant differences using a rank sum test (Rtn4 vs. Sec61β: P <<< 0.001; Rtn4 vs. KDEL: P <<< 0.001; Sec61β vs. KDEL: P <<< 0.001). **(B)** Polar histogram of φ from analysis using the nanobody localizations of Sec61β. There were 765,458 unique angle calculations total across the *n* = 3 biological replicates, respectively. **(C)** Polar histogram of φ from analysis using the nanobody localizations of KDEL. There were 889,347 unique angle calculations total across the *n* = 3 biological replicates, respectively. **(A–C)** *n* = 3 cells from unique sample preparations; error bars represent the standard deviation of the frequency of each histogram bin between biological replicates.

In our 3D data, Rtn4 also appeared to prefer the sides of tubules, in the x-y plane of the sample, over the tops and bottoms of them (Fig. 2 A), supporting a recently reported observation (Wang et al., 2022). Most ER models depict tubules with circular cross-sections (Goyal and Blackstone, 2013; Georgiades et al., 2017; Shibata et al., 2010; Wang and Rapoport, 2019; Chen et al., 2013; Lin et al., 2012; Shemesh et al., 2014; Obara et al., 2022; Hu et al., 2008), and one would expect a random distribution of Rtn4 around such tubules. However, as recently suggested (Wang et al., 2022), tubules with elliptical cross-sections, where Rtn4 preferentially locates to the regions of higher membrane curvature, could explain our observation. To investigate this in more detail, we considered four possible models (Fig. 3 A). To test which of these models is consistent with our data, we used 3D-SMLM to investigate the spatial distribution of Rtn4, Sec61β, and KDEL in tubule cross-sections. Each sample was labeled with a nanobody, whose localizations were used to generate 3D surfaces, and primary and secondary antibodies, whose localizations were used to analyze the proteins’ spatial distribution. The 3D surfaces were subsequently used to create “skeletons” of the ER networks by applying mean curvature flow (Tagliasacchi et al., 2012; Fig. S2 E; see Materials and methods). These skeletons provide critical information about the center and major axis of each tubule in the datasets (Fig. 3, B–D). Using this information, we calculated the angular position of each protein in tubule cross-sections relative to the z-axis of the sample (normal to the coverslip). We refer to these angles as the cross-sectional angles φ (Fig. 3 A; model [i]). We compared the distributions of φ for the three proteins to that of simulated points randomly distributed in a circle around the skeletons (Fig. 3, E–G and Fig. S2 F). We found that Rtn4, Sec61β, and KDEL are all enriched around ±90° from the z-direction of the cross-sections when compared with simulated random distributions. To show that these results are not an artifact caused by the large size of primary and secondary antibodies, we repeated the φ analysis on the nanobody localizations of Sec61β and KDEL and observed similar results (Fig. S3, B and C). Since KDEL is localized to the ER lumen and no preference of Sec61β for specific membrane topology has been reported, we conclude from this observation that ER tubules are on average elliptical in shape, ruling out the first two models. Additionally, the three φ distributions are significantly different from each other with Rtn4 being the most enriched at the sides, followed by Sec61β and then KDEL (Fig. S3 A). The only model that is consistent with these observations is model (iv; Fig. 3 A), i.e., tubules with elliptical cross-sections and Rtn4 localized to the sides where the local curvature is highest. Indeed, visualizing cross-sections of tubules in our two-color 3D-SMLM data of Rtn4 and Sec61β revealed cross-sections with elliptical shapes and distributions of these two proteins, consistent with model (iv; Fig. 3, H–N).

### Rtn4 oligomers contain an average of five copies

**Figure S4.**
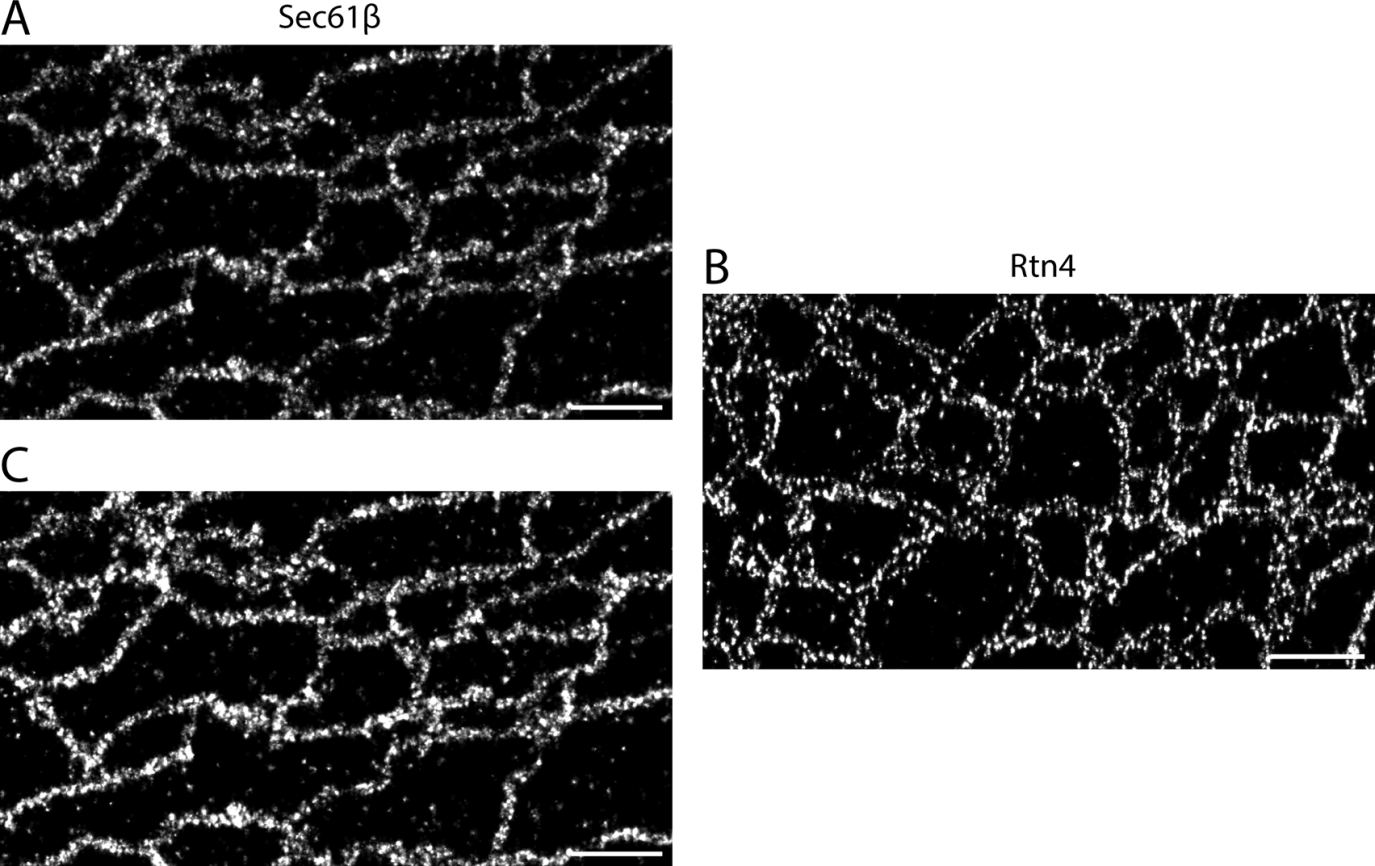
**Comparison between Sec61β and Rtn4 localizations. (A and B)** Sec61β and Rtn4 displayed with identical contrast settings. **(C)** The same image as A but displayed with adjusted contrast so that it can be more easily compared with B. **(A–C)** Scale bars represent 1 µm.

**Figure 4.**
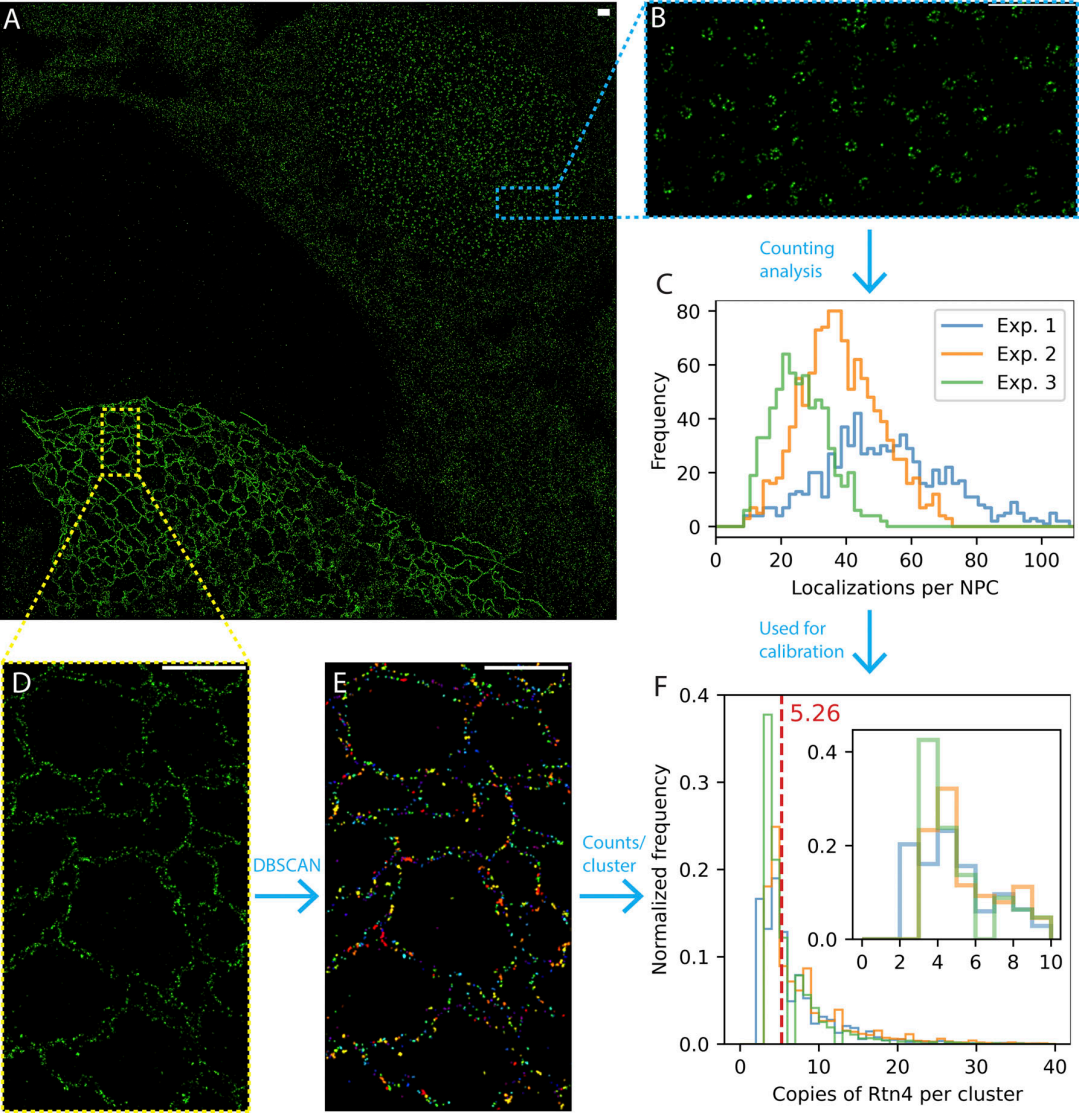
**Counting the number of Rtn4 copies per oligomer. (A)** Representative whole field-of-view of a dataset. A U-2 OS Rtn4-mEGFP cell and a U-2 OS Nup96-mEGFP cell are located in the bottom left and the upper right of the image, respectively. **(B)** Zoom-in of Nup96-mEGFP. **(C)** Histograms from each experiment of the number of localizations per nuclear pore complex. **(D)** Zoom-in of Rtn4-mEGFP. **(E)** Same zoom-in as D after segmenting clusters of Rtn4 localizations using DBSCAN. Clusters are colored uniquely. **(F)** Histograms from each experiment (experiment #1: blue, experiment #2: orange, experiment #3: green) of the number of Rtn4 copies per cluster of Rtn4 localizations. Median values of Rtn4 copies per cluster—experiment #1: 5.36, experiment #2: 5.67, experiment #3: 4.75. Inset shows the same plot with the x-axis limited to 10 copies of Rtn4 per cluster. Scale bars represent 1 μm.

A visual comparison of Sec61β and Rtn4 point clouds from our 3D SMLM data of ER tubules clearly shows Rtn4 being clustered while Sec61β appears diffusely localized (Fig. S4), supporting earlier reports of Rtn4 oligomerization (Shibata et al., 2008). To gain insight into the organization of individual Rtn4 oligomers, we determined how many copies are in a typical oligomer using the powerful SMLM variant of fluorogenic DNA-PAINT (Chung et al., 2022) and single-molecule counting. Following an established procedure (Thevathasan et al., 2019), we used nucleoporin Nup96 as a standard to resolve the complications of single molecule counting (Baddeley and Bewersdorf, 2018). We mixed a U-2 OS Nup96-mEGFP cell line (Thevathasan et al., 2019) with a U-2 OS Rtn4-mEGFP cell line (Fig. S1, D–F), both endogenously tagged using CRISPR and including all isoforms of Rtn4, into one sample and labeled the proteins of interest with an anti-GFP nanobody conjugated to a docking strand for fluorogenic DNA-PAINT probes. This allowed us to image both Nup96 and Rtn4 in one field of view, ensuring that all sample preparation and imaging conditions between both proteins were identical (Fig. 4 A). Using the results of the Nup96-mEGFP counting analysis (Fig. 4, B and C; Thevathasan et al., 2019) to calibrate the DBSCAN (Ester et al., 1996; Schubert et al., 2017) segmented clusters of Rtn4-mEGFP (Fig. 4, D and E) showed that a typical cluster of Rtn4-mEGFP contains approximately five copies of the protein (average of the median values across three experiments: 5.26; Fig. 4 F, red dashed line). This result is consistent with previously reported in vitro studies (Shibata et al., 2008) and confirms that Rtn4 also forms oligomers around this size in situ.

### Rtn4 forms linear-shaped clusters enriched at orientations near parallel to the tubule axis

**Figure 5.**
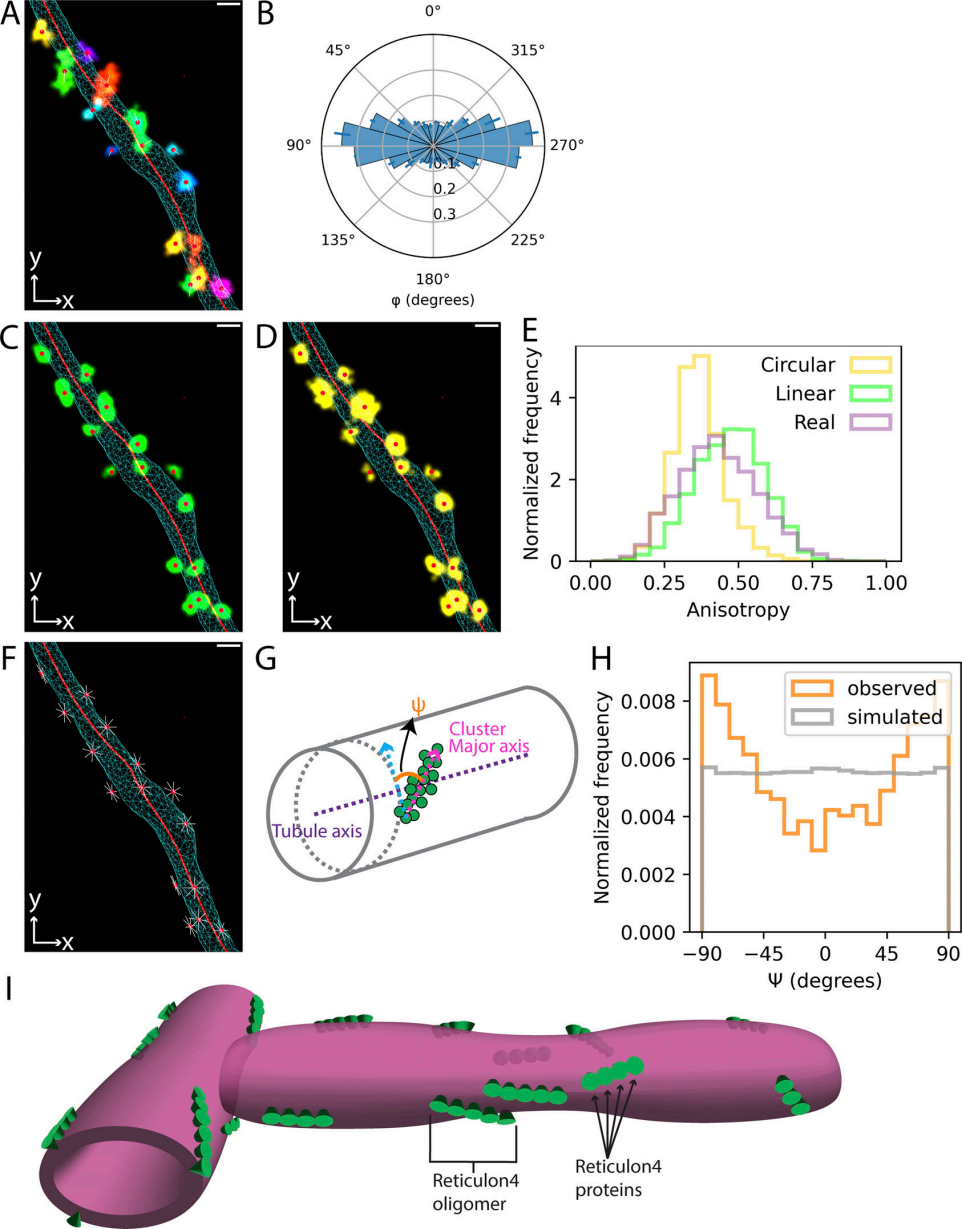
**Position, shape, and orientation of Rtn4 oligomers. (A)** An ER tubule showing the 3D surface as a cyan wireframe, the skeleton as a red line, each observed Rtn4 cluster colored uniquely, the centers of clusters as red circles, and the major axis of each cluster as a white line. **(B)** Polar histogram of φ for the centers of Rtn4 clusters (*n* = 3 cells from unique sample preparations; Error bars represent the standard deviation of the frequency of each histogram bin between biological replicates). The data represents 11,427 unique angle calculations total across the *n* = 3 biological replicates. **(C)** Similar to A but with linear-shaped simulated clusters (green) instead of observed clusters. **(D)** Similar to A but with circular-shaped simulated clusters (yellow) instead of observed clusters. **(E)** Histograms of the anisotropy of observed clusters (lavender), linear-shaped simulated clusters (green), and circular-shaped simulated clusters (yellow). **(F)** Similar to A excluding observed clusters and with simulated cluster major axes shown that are randomly oriented and tangential to the tubule surface. **(G)** Model of the vectors that ψ is being calculated between. ψ is calculated between the major axes of the clusters (magenta arrow) and vectors orthogonal to the tubule axis (blue arrow). See Materials and methods for more details. **(H)** Histogram of ψ for observed clusters and clusters simulated to possess all orientations tangential to the tubule surface (F). **(I)** Model of Rtn4 organization and its effects on tubule structure. Individual Rtn4 proteins are depicted as green cones on a magenta tubule surface.

**Figure S5.**
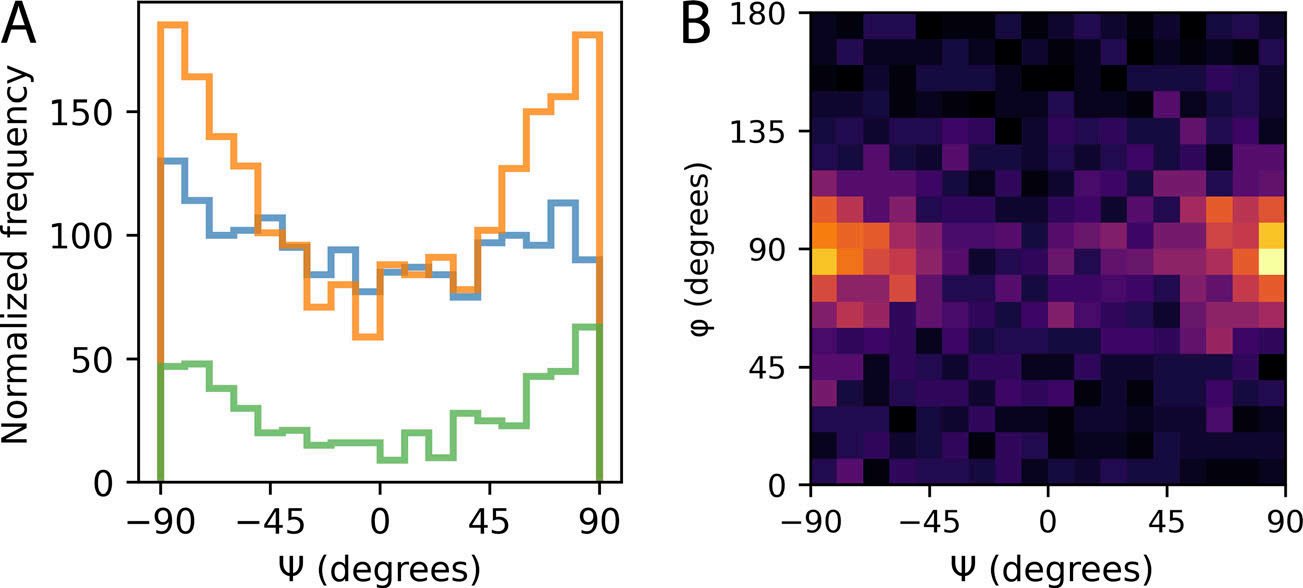
**Distribution of different sized clusters’ ψ and how ψ and φ relate. (A)** Histograms of ψ for small (blue; <10 localizations), medium (orange; 10 < localizations < 50), and large (green; >50 localizations) clusters. **(B)** 2D histogram of ψ vs. φ.

We next sought to understand how Rtn4 oligomers are organized in the ER membrane. We used DBSCAN on the 3D Rtn4 point clouds to segment the localizations into clusters (Fig. 5 A and Video 2). Analysis of φ for the centers of each cluster (Fig. 5 B) showed a similar result to the angular distribution of the Rtn4 localizations directly (Fig. 3 E). We next used principal component analysis (PCA; Jollife and Cadima, 2016) to determine the principal axis of each cluster with the highest eigenvalue, which we refer to as the major axis. PCA also allowed us to analyze the shape of Rtn4 clusters by comparing the anisotropy of observed clusters to simulated clusters that were circular- or linear-shaped (Fig. 5, C and D; and Videos 3 and 4). The observed clusters had anisotropy distributions much more similar to the linear-shaped simulated clusters than the circular-shaped ones (Fig. 5 E). This suggests that the Rtn4 oligomers are organized linearly with discernable major axes. The clusters clearly did not consistently appear as arches oriented orthogonal to the tubule axis (Shibata et al., 2008; Hu et al., 2008), which led us to ask if the clusters’ major axes adopted consistent orientations relative to the tubule axes. To help answer this question, we simulated clusters with randomly oriented major axes that are tangential to the ER surface (Fig. 5 F and Video 5). We further projected the vectors of the major axes of the observed clusters onto a plane approximately tangential to the 3D surface (Fig. 5 G; red vector) and calculated the angles ψ between them and vectors in the same plane but perpendicular to the tubule axis (Fig. 5 G; blue vector). Comparing the distributions of ψ for the observed and simulated clusters shows that the observed clusters are strongly enriched at orientations close to parallel with the tubule axis (±90°) and depleted at orientations close to orthogonal to the tubule axis (Fig. 5 H). This orientation preference of the observed clusters only disappears when limiting the analysis to the smallest clusters where the cluster orientations cannot be resolved by our microscope (Fig. S5 A). Plotting φ against ψ, we also see that the orientation of the clusters does not depend on where the clusters are located on the tubules (Fig. S5 B). Our findings in this study culminate in a refined model of Rtn4 organization, localization, and the resulting shape of ER tubules (Fig. 5 I).

**Video 2.** **Rtn4 clusters.** U-2 OS cells transiently transfected with mCherry-Sec61β imaged with 3D-SMLM on our 4Pi-SMLM microscope. A single tubule taken from the dataset in Fig. 2 displaying the surface (cyan), skeleton (red line), the center of each cluster (red circle), the major axis of each cluster (white line), and clusters of Rtn4 points (varying colors). Scale bar represents 50 nm. The playback speed is 60 frames per second.

**Video 3.** **Circular simulated clusters.** U-2 OS cells transiently transfected with mCherry-Sec61β imaged with 3D-SMLM on our 4Pi-SMLM microscope. A single tubule taken from the dataset in Fig. 2 displays the surface (cyan), skeleton (red), the center of each cluster (red circle), and circular simulated clusters (yellow). Scale bar represents 50 nm. The playback speed is 60 frames per second.

**Video 4.** **Linear simulated clusters.** U-2 OS cells transiently transfected with mCherry-Sec61β imaged with 3D-SMLM on our 4Pi-SMLM microscope. A single tubule taken from the dataset in Fig. 2 displays the surface (cyan), skeleton (red), the center of each cluster (red circle), and linear simulated clusters (green). Scale bar represents 50 nm. The playback speed is 60 frames per second.

**Video 5.** **Simulated cluster orientations.** U-2 OS cells transiently transfected with mCherry-Sec61β imaged with 3D-SMLM on our 4Pi-SMLM microscope. A single tubule taken from the dataset in Fig. 2 displaying the surface (cyan), skeleton (red), the center of each cluster (red circle), and the orientations of simulated clusters possessing all possible orientations tangential to the surface (white lines; only a subset of all the orientations is displayed). Scale bar represents 50 nm. The playback speed is 60 frames per second.

With elliptical tubules, the line between what is a tubule and what is a sheet becomes blurred. Recent studies of ER structure have made it clear that this differentiation is not as binary as once thought. Instead, the ER displays a spectrum of morphologies (Nixon-Abell et al., 2016; Schroeder et al., 2019; Puhka et al., 2012). Thus, it may be more appropriate to think of the ER as being composed of a spectrum of sheets including continuous sheets, fenestrated sheets, and now (structurally speaking) very narrow sheets, referred to as ribbon-like ER sheets by Wang et al. (2022), that have traditionally been thought of as tubules.

We consistently observed the major axes of elliptical tubule cross-sections lying in the x–y plane. We do not believe this to be universally true, but rather a side effect of focusing our imaging efforts in the lamellipodia of very flat cells restricting the tubules.

It was recently suggested that ER tubules exist in two distinct forms with consistent diameters of ∼105 and ∼50 nm, with U-2 OS cells’ tubules existing predominantly in the ∼105-nm form (Wang et al., 2022). However, our diameter measurements here and in previous analyses (Barentine et al., 2018; Schroeder et al., 2019) do not show consistent narrowly distributed tubule measurements with peaks around these values. Instead, they show broad ranges that include tubules with ∼105 and ∼50 nm amongst many other sizes. However, this may be explained by differing methods. Our approach used Sec61β as an ER membrane proxy since it is a better general ER membrane marker than Rtn4 and an established tool to measure the tubule diameters (Barentine et al., 2018).

**Figure S6.**
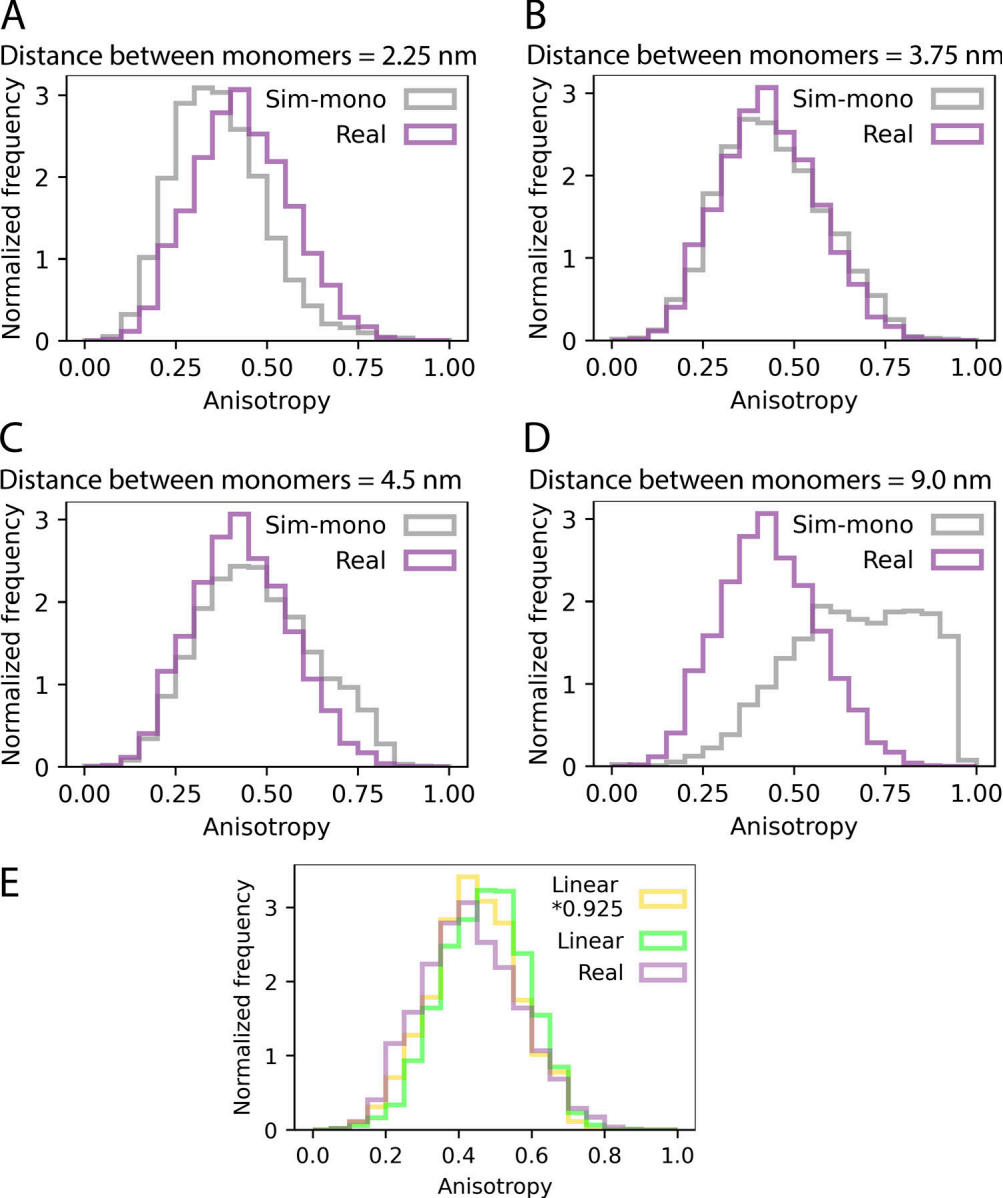
**Anisotropy histograms of simulated clusters. (A–D)** Histograms of observed cluster anisotropies (lavender) and clusters simulated with specific distances between the Rtn4 monomers (gray). **(E)** Anisotropy distributions of observed clusters (lavender), linear-shaped clusters using the radius of gyration (Rg) of observed clusters (green), and linear-shaped clusters using Rg * 0.925 (yellow).

We have also shown that Rtn4 organizes into oligomers of around five copies of the protein. However, it is not strictly a pentamer. As our results show, there is a distribution of sizes. To get a sense of how monomers of Rtn4 are organized within an oligomer, we simulated point clouds of Rtn4 oligomers possessing specific distances between monomers positioned in a line (see Materials and methods) and compared their anisotropies to those of observed Rtn4 oligomers (Fig. S6). We calculated the theoretical diameter of Rtn4b molecules to be ∼4.5 nm (see Materials and methods), assuming they are roughly spherical. Using this value as the intermonomer distance in the simulation resulted in remarkably similar distributions between the observed and simulated clusters’ anisotropies (Fig. S6 C), while simulations for half or double that distance showed strong discrepancies (Fig. S6, A and D). Decreasing the intermonomer distance to 3.75 nm resulted in a nearly perfect overlap between simulation and experimental results (Fig. S6 B). This simple simulation supports the idea that Rtn4 oligomers are composed of monomers that roughly organize in a linear fashion. Additionally, using this theoretical ∼4.5 nm diameter and the surface area of our 3D surfaces, we estimate that Rtn4 proteins occupy about 4% of the ER membrane surface area (see Materials and methods).

Until now, it was attractive to think that Rtn4 oligomers formed arches around tubules and sheet edges to scaffold the membrane into a curvature (Shibata et al., 2008; Hu et al., 2008). However, we have seen the majority of oligomers at orientations near parallel to tubule axes, which is the opposite of what one would expect for arching oligomeric scaffolds. Our results are also inconsistent with the possibility of Rtn4 forming splayed dimers (Wang et al., 2021). We, therefore, believe that the oligomerization of Rtn4 in and of itself does not contribute to membrane curvature and instead serves as a mechanism to increase local Rtn4 concentration, which our results show correlates with higher curvature. This further supports a model in which the membrane curvature is primarily induced by a hairpin wedging mechanism (Zurek et al., 2011; Voeltz et al., 2006). This explains both the importance of Rtn4’s oligomerization and the lack of its oligomers orienting in a way that would be amenable to a scaffolding mechanism. Consistent with this idea, long transmembrane domain mutants of Rtn4’s RHD have been reported to still form oligomers while lacking the ability to curve the ER membrane (Zurek et al., 2011). We assume that the membrane curvature generated by Rtn4 monomers is in a single direction and that they are oriented in a specific direction within oligomers. Considering that oligomers preferentially orient parallel to the tubule axis, this model suggests that Rtn4 monomers generate curvature in the direction perpendicular to the major axis of oligomers.

Given Rtn4’s shared characteristics with other known ER membrane-curving proteins (Zurek et al., 2011; Voeltz et al., 2006; Shibata et al., 2008; Hu et al., 2008), it is reasonable to think that this model could be generalized to all RHD-containing proteins as they seem to have some functional redundancy. However, this still needs to be confirmed experimentally and is an interesting future direction.

## Materials and methods

### Cell culture

U-2 OS (HTB-96; Lot #70008732; ATCC) were cultured in McCoy’s 5A (16600-082; Gibco) + 10% FBS (10438-026; Gibco). U-2 OS Rtn4-mEGFP, U-2 OS Rtn4-Halo, and the U-2 OS Nup96-mEGFP (product number: 300174; Cell Lines Service) CRISPR cell lines were cultured in the same way. Subculturing was performed using 0.05% Trypsin (25200056; Gibco). Transfections were performed using a Super Electroporator NEPA21 Type II (Nepa Gene). Cells were concentrated to 1 million cells in 90 μl in OptiMEM (31985088; Gibco; Thermo Fisher Scientific) in an electroporation cuvette (12358-346; Bulldog Bio) to which 10 μl of ∼1 μg/μl of plasmid DNA was added. Cells were electroporated using the following settings: poring pulse: 125 V, 3 ms pulse length, 50 ms pulse interval, 2 pulses, 10% decay rate, and + polarity; transfer pulse: 25 V, 50 ms pulse length, 2 pulses, 40% decay rate, and ± polarity. Prior to seeding cells, the coverslips for fixed samples were treated with ozone for 30 min, the ibidi slides for DNA PAINT were cleaned with plasma for 5 min (PDC-001; Harrick Plasma), and the dishes for live cells were cleaned by placing them in KOH and sonicating them in a water bath for 15 min. Cells were then seeded onto 30-mm round coverslips (30-1313-03192; Bioptechs) for fixed 4Pi-SMS samples, ibidi slides (80607; ibidi) for fluorogenic DNA PAINT single-molecule counting, or MatTek dishes for live STED samples (P35G-1.5-20-C; MatTek). Cells were allowed to adhere to the coverglass and reach desirable transient expression levels for 16–24 h after transfection before proceeding with sample preparation.

### Plasmids

mEmerald-Sec61β and mCherry-Sec61β were acquired from Addgene (plasmids 54249 and 49155, respectively). GFP-KDEL was cloned as previously described (Merta et al., 2021). In brief, a pDsRed2-ER vector (632409; Clontech) was digested with AgeI and HindIII to remove DsRed2, and a PCR-amplified GFP sequence with AgeI and HindIII sites was ligated into the vector. SNAP-Sec61β and Halo-Sec61β were cloned as previously described (Bottanelli et al., 2016). Briefly, mEmerald-Sec61β (above) was digested with NheI and BgIII to remove mEmerald, and a PCR-amplified HaloTag or SNAP-tag sequence with NheI and BgIII sites was ligated in mEmerald’s place.

### CRISPR gene editing

The U-2 OS Rtn4-Halo cell line was developed using Cas9 gene editing. Though we expect U-2 OS cells to have high expression of Rtn4b and little to no expression of the other isoforms (Oertle and Schwab, 2003; Uniprot Q9NQC3), we approached the CRISPR gene-editing in a way that would tag all isoforms. Since all Rtn4 isoforms share the same C-terminus, we opted to add the HaloTag to the C-terminus. This ensures that all Rtn4 isoforms expressed in U-2 OS cells will possess the appropriate tag. A gRNA described previously to generate a U-2 OS Rtn4-SNAP cell line was used in the generation of this cell line as well (5′-AAACGCCCAAAATAATTAGTAGG-3′; the PAM site is underlined; Schroeder et al., 2019). The homology-dependent repair (HDR) template, containing ∼1 kb homology arms from that study, was modified to replace the SNAP-tag sequence with the HaloTag sequence. This was accomplished by using a two-fragment Gibson Assembly using a NEBuilder reaction (E5520S; New England Biolabs). The fragments were amplified using PCR with the following primers (uppercase indicates Gibson overlapping bases):

Halo-Forward: 5′-GTC​GCC​ACC​ATG​GCA​GAA​ATC​GGT​ACT​GG-3′
Halo-Reverse: 5′-GCT​TTA​GCC​GGA​AAT​CTC​GAG​CGT​C-3′
Vector-Forward: 5′-CGA​GAT​TTC​CGG​CTA​AAG​CGG​CCG​CGA​CTC​TAG​ATC-3′
Vector-Reverse: 5′-ATT​TCT​GCC​ATG​GTG​GCG​ACC​GGT​GGA​TC-3′.

U-2 OS (HTB-96; Lot #70008732; ATCC) were transfected with the gRNA, HaloTag HDR template, and pSpCas9 (48137; Addgene) via Lipofectamine 2000 in the well of a six-well plate. Transfected cells were expanded for 1 wk before selection with G418 began. After 9 d of selection, cells were labeled with SiR-chloroalkane for 1 h followed by 3× washes with warm media and a 1-h recovery at 37°C. The cells were immediately sorted using FACS to obtain monoclonal cell lines. Cells were first screened visually for fluorescence consistent with Rtn4 localization. Then the success and zygosity of gene editing were assessed by Western blotting.

The U-2 OS Rtn4-mEGFP cell line was developed using Cas9 nickase D10A to increase the chances of developing a homozygously tagged cell line (Koch et al., 2018). For the reasons described above, we added the mEGFP tag to the C-terminus of Rtn4. Following the protocol from Koch et al. (2018), a plasmid was cloned via T4 ligase using annealed oligos for the paired gRNA sequences and pX335-U6-Chimeric_BB-CBh-hSpCas9n(D10A) plasmid (Addgene; 42335) cut with BbsI restriction enzyme. The gRNA sequences were as follows: sense: 5′-AAACGCCCAAAATAATTAGT-3′; anti-sense: 5′-CAGCTTTGCGCTTCAATCCA-3′. An HDR template vector containing 800 bp homology arms flanking the RTN4 locus (Gene ID: 57142) was ordered from Genewiz. The mEGFP tag was cloned into the vector using a two-fragment Gibson assembly with the following primers (uppercase indicates Gibson overlapping bases):

mEGFP-Forward: 5′-GGT​GAG​CAA​GGG​CGA​GGA​GCT​GTT​CAC-3′
mEGFP-Reverse: 5′-GGC​GTT​TTC​ACT​TGT​ACA​GCT​CGT​CCA​TGC-3′
Donor-Vector-Forward: 5′-GCT​GTA​CAA​GTG​AAA​ACG​CCC​AAA​ATA​ATT​AGT​AG-3′
Donor-Vector-Reverse: 5′-GCT​CCT​CGC​CCT​TGC​TCA​CCA​TGG​TGG-3′.

U-2 OS (HTB-96; Lot #70008732; ATCC) were transfected with the pX335-U6-Chimeric-sense-gRNA, pX335-U6-Chimeric-antisense-gRNA, and mEGFP HDR template plasmids using JetPrime (114-07; Polyplus transfection). Cells were expanded for 6 d before sorting with FACS to obtain monoclonal cell lines. Cells were expanded after FACS for 3 wk before visually screening for fluorescence consistent with Rtn4 localization. The success and zygosity of gene editing were assessed by Western blotting.

### Western blotting

U-2 OS, U-2 OS Rtn4-Halo, U-2 OS Rtn4-mEGFP, and U-2 OS Rtn4-Knockout (KO) cells were seeded into the wells of six-well plates (∼350,000 cells per well) and were allowed to adhere to the wells overnight at 37°C. The following day, media was aspirated from the wells of the 6-well plate, and a buffer containing 900 μl of 4× Laemmli Sample Buffer (1610747; Bio-Rad) and 100 μl of β-mercaptoethanol was added to each well (100 μl per well). Cells were then scraped off the bottom of the well using a cell scraper (353086; Falcon) and pipetted into unique tubes. Samples were run on NuPAGE 4–12% Bis-Tris Gels (NP0335BOX; Invitrogen) in MES SDS buffer for 1 h at 120 V. Proteins were then transferred to a PVDF membrane at 30 V for 1 h at 4°C in 1× transfer buffer (25 mM Tris base, 19 mM glycine, and 20% methanol). Membranes were blocked in block buffer (5% [w/v] milk in PBS-T) for 1 h at room temperature. Then, membranes were incubated with primary antibodies diluted in block buffer (rabbit anti-Rtn4 at 1:1,000: ab47085; Abcam; rabbit anti-Halo at 1:100: G928A; Promega; Rabbit anti-GFP at 1:100: A11122; Invitrogen; mouse anti-alpha-tubulin at 1:3,000: ab89984; Sigma-Aldrich) overnight at 4°C. The following day, membranes were washed 3× for 5 min each with PBS-T followed by incubation with secondary antibodies diluted in block buffer (goat anti-rabbit-HRP at 1:2,000: 7074S; Cell Signaling Technology; horse anti-mouse-HRP at 1:2,000: 7076S; Cell Signaling Technology) for 1 h at room temperature. Membranes were washed again 3× for 5 min each. Clarity Western ECL Substrate (Bio-Rad) was added onto the membranes which were imaged using an ImageQuant LAS 4000 with chemiluminescence and 10-s to 1-min exposures.

### Immunofluorescence

U-2 OS cells (HTB-96; Lot #70008732; ATCC) or cell lines generated from this cell line, other than U-2 OS Nup96-mEGFP, were used for every experiment. All incubations for immunofluorescence samples were done at room temperature with sample rocking, unless otherwise noted. Cells were fixed in 3% paraformaldehyde +0.1% glutaraldehyde in 1× PBS for 15 min. The fixation was then quenched with 0.1% sodium borohydride in 1× PBS for 7 min followed by 100 mM glycine in 1× PBS for 10 min. Samples were rinsed three times with 1× PBS and permeabilized with room temperature permeabilization buffer (0.3% IGEPAL CA-630 [I8896; Sigma-Aldrich], 0.05% Triton X-100 [T8787; Sigma-Aldrich], 0.1% BSA [001-000-162; Jackson ImmunoResearch] in 1× PBS) for 3 min followed by another three washes with 1× PBS. Samples were blocked for 1 h with block buffer (0.05% IGEPAL CA-630, 0.05% Triton X-100, 5% goat normal serum [005-000-121; Jackson ImmunoResearch] in 1× PBS). Samples were incubated with primaries/nanobodies diluted in block buffer for 16 h at 4°C on a rocker. Excess primaries/nanobodies were washed away with three 5-min incubations with wash buffer (0.05% IGEPAL CA-630, 0.05% Triton X-100, 0.2% BSA in 1× PBS) before incubating samples with secondaries diluted in block buffer for 1 h. Excess secondaries were washed away with three 5-min incubations with wash buffer. Finally, samples were rinsed three times with 1× PBS. 4Pi-SMS samples were post-fixed with 3% paraformaldehyde + 0.1% glutaraldehyde in 1× PBS for 10 min followed by three rinses with 1× PBS.

### Preparation for STED imaging

For live cell STED imaging of U-2 OS Rtn4-Halo cells transiently overexpressing SNAP-Sec61β, cells were live labeled with 1 µM ATTO 590-chloroalkane and 1 µM of SNAP-Cell 647-SiR (S9102S; New England Biolabs; final concentrations) diluted in warm media for 1 h at 37°C. For the live cell STED imaging, U-2 OS and U-2 OS Rtn4-knockout cells both transiently overexpressing Halo-Sec61β were live-labeled with 1 µM ATTO 590-chloroalkane diluted in warm media for 1 h at 37°C. All live cell STED samples were rinsed three times with warm media and allowed to recover for 1 h at 37°C following incubation with diluted dyes. The media was replaced with Live Cell Imaging Solution (A14291DJ; Gibco) supplemented with 15 mM glucose (G5767; Sigma-Aldrich). Cells were then imaged immediately while being maintained at 37°C with 5% CO_2_.

### Preparation for single-molecule counting

U-2 OS Rtn4-mEGFP and U-2 OS Nup96-mEGFP cells were removed from flasks using trypsin, mixed together, and seeded into the lanes of a plasma cleaned ibidi slide. The next day, cells were fixed and labeled as described above for immunofluorescence. The samples were labeled with a custom-ordered anti-GFP nanobody conjugated to a single docking site for fluorogenic DNA PAINT imaging (Chung et al., 2022; Massive Photonics). After washing out excess nanobody from the overnight labeling, samples were imaged immediately using 50 nM of Cy3B imager B in a PBS-based buffer (1× PBS, 500 mM NaCl, 1 mM Trolox, 20 mM sodium sulfite, pH 7.3–7.5). Trolox was stored in 1 M aliquots at −20°C in DMSO.

### Preparation for 4Pi-SMS imaging

The following samples were labeled sequentially starting with the nanobody overnight at 4°C, then the primary antibody overnight at 4°C, and finally the secondary antibody for 1 h at room temperature. U-2 OS transiently overexpressing mCherry-Sec61β were prepared for immunofluorescence as described above using a rabbit anti-Rtn4 primary (ab47085; Abcam) with a goat anti-rabbit secondary conjugated to Alexa Fluor 647 (A21245; Life Technologies) and a custom ordered FluoTag X4 anti-RFP nanobody conjugated to CF660C (N0404; NanoTag). U-2 OS transiently overexpressing GFP-KDEL or mEmerald-Sec61β were both prepared for immunofluorescence as described above using a rabbit anti-GFP primary (A-11122; Invitrogen) with a goat anti-Rabbit secondary conjugated to CF660C (20812; Biotium), and a FluoTag X4 anti-GFP nanobody conjugated to Alexa Fluor 647 (N0304; NanoTag). To enable microscope alignment, 100-nm crimson Fluospheres (F8816; Thermo Fisher Scientific) were added on top of all samples. Mounting the samples was performed as reported previously (Zhang et al., 2020). Briefly, STORM imaging buffer was prepared fresh just before imaging (143 mM β-mercaptoethanol, 50 mM Tris pH 8.0, 50 mM NaCl, 10% glucose, 135 U/μl catalase, and 1 U/μl glucose oxidase). Samples were mounted onto a custom-made sample holder facing up. Crimson Fluospheres (F8806; Thermo Fisher Scientific) were added onto the coverslip and were allowed to settle for approximately 5 min before removing the excess buffer. Then, 100 μl of STORM buffer was added to the coverslip, and a second coverslip was added on top of it. Excess STORM buffer was removed using Kimwipes, and the coverslips were sealed with two-component glue (Picodent Twinsil, Picodent) which was allowed to solidify for ∼20 min.

### Microscopy

STED imaging was performed using a Leica SP8 STED 3× using a pulsed white light laser for excitation (SuperK Extreme EXW-12; NKT Photonics) and a pulsed 775 nm laser for depletion (Onefive Katana-08HP). A 100 × 1.40 NA-high contrast Plan Apochromat oil CS2 objective was used for all image acquisition using the Application Suite X Software (LAS X; Leica Microsystems). ATTO 590-chloroalkane and SNAP-Cell 647-SiR present in the same sample were imaged using excitation wavelengths of 592 nm (∼26 μW) and 650 nm (∼11 μW), respectively, and 775 nm (∼28 mW) depletion wavelength. HyD hybrid detectors with 0.3–6.0 ns gating were used to record fluorescence ranging from 600–630 nm for ATTO 590 and 650–750 nm for SNAP-Cell 647-SiR. Images were acquired sequentially between lines starting with SNAP-Cell 647-SiR using eight line-average and 8,000 Hz resonant scanning. One-color imaging of ATTO 590 in live cells was performed using 592 nm excitation at 24 µW, 775 nm depletion at 49 mW, 604–700 nm detection using a HyD hybrid detector with 0.3–6.0 ns gating, 8 line-average, and 8,000 Hz scanning. Cells in all samples were maintained at 37°C and 5% CO_2_. For figures, STED images were convolved with a Gaussian blur with a Sigma-Aldrich of 1 pixel using ImageJ software. All analyses performed on STED images were done on the raw images.

4Pi-SMLM samples labeled with CF660C and Alexa Fluor 647 were imaged with a ratiometric approach using salvaged fluorescence on a 4Pi-SMLM microscope. The localization analysis and drift correction were executed in custom MATLAB scripts. All of this was performed as described previously (Zhang et al., 2020; Huang et al., 2016). The 4Pi-SMLM microscope possesses opposing silicon oil immersion objectives (100× 1.35 NA; Olympus). A 642-nm laser was used for excitation (2RU-VFL-P-2000-642-B1R; MPB Communications) and a 405-nm laser was used for activation (OBIS 405 LX, 50 mW; Coherent). The conventional fluorescence was recorded on a sCMOS camera (ORCA-Flash 4.0v2; Hamamatsu). The salvaged fluorescence was recorded by an EMCCD camera (128 × 128 pixels, iXon DU860; Andor). The microscope hardware was controlled by custom-written LabVIEW software (International Instruments). Samples were imaged at room temperature in STORM buffer (described above).

Single-molecule counting samples were imaged using Fluorogenic DNA PAINT on a Nikon Eclipse Ti2 microscope (Nikon Instruments) with an attached Andor Dragonfly 500 unit (Andor, Oxford Instruments). Cy3B conjugated to fluorogenic imager probe B (Chung et al., 2022) at a working concentration of 50 nM was excited using a 561-nm laser with an 8.2 × 8.2 mm illumination aperture and a P2 power density filter resulting in a laser intensity of ∼1 mW/cm^2^. Fluorescence was filtered through a TR-DFLy-F600-050 emission filter before being collected using a 1.49 NA 60× oil TIRF objective and captured on a Sona 4BV6X sCMOS camera (Andor Technologies) on 512 × 512 pixels with an effective pixel size of 108 nm at 20 Hz. The microscope was controlled using Fusion Software (Andor). Samples were imaged at room temperature.

### ATTO 590**–**Chloroalkane probe preparation

HaloTag chloralkane amine (2.2 mg, 9.9 µmol, 3.0 equiv) was prepared as previously described (Tyson et al., 2021) and added to a mixture of ATTO590-NHS (2.5 mg, 3.3 μmol, 1.0 eq) and iPr2NEt (5.5 μl, 33 μmol, 10 eq) in DMSO. The reaction mixture was stirred for 3 s under the exclusion of light. Following the completion of the reaction as tracked by LC-MS, the reaction mixture was purified directly via reverse-phase HPLC. The title compound was obtained as a solid after evaporation under reduced pressure of the collected HPLC fractions. HRMS(ESI): m/z calc. for C47H59ClN3O6+: 796.4087; found: 796.4073. UPLC:tR = 1.88 min; gradient: 0 min: 5% B, 1 min: 5% B, 1.6 min: 95% B, 3.0 min: 95% B; C18 column.

### Skeleton generation and tubule major axis determination

To generate skeletons from a surface, we implemented a mean curvature skeletonization (Tagliasacchi et al., 2012) approach in PYMEVisualize (Marin et al., 2021). We used a weighting of 20 and 0.01 for the velocity and medial axis terms, respectively, to create the initial mesoskeleton. Then, the position of each point on the skeleton was changed to the average position of all points within 50 nm of that point. We applied an upper threshold of 200 to the density of skeleton points within 50 nm of any given skeleton point. Points exceeding this threshold were removed from the skeleton. This served to remove parts of the skeletons found within ER sheets, so analyses were restricted to tubules only. The major axis of the tubule at any given skeleton point was determined by calculating vectors pointing from each skeleton point to the nearest skeleton point that was a minimum of 10 nm away.

### Data analysis

#### Rtn4 pixel intensity

The average Rtn4 intensity per pixel along tubules was analyzed using ImageJ by creating 10-pixel-wide regions of interest (ROI) along tubules. The Rtn4 pixel intensities were summed across the width of the line plots and those sums were averaged along the length of the ROI. The two populations of Rtn4 pixel values were tested for a statistically significant difference by computing the Wilcoxon rank-sum statistic.

#### Tubule diameter comparison

Tubule diameters were measured using 10-pixel wide line plots in Python Microscopy Environment (PYME) with the NEP fitting plug-in (Barentine et al., 2018). NEP fitting uses ensemble model-based fitting to extract accurate diameters and resolutions for STED-imaged ER tubules, amongst other structures. The “STEDTubule_SurfaceSNAP” model was used for all tubule diameter measurements. Pairs of tubule diameters populations were tested for statistically significant differences by computing the Wilcoxon rank-sum statistic.

#### Mean curvature and Rtn4 density analysis

3D surfaces of the ER were created in PYMEVisualize using the NanoWrap algorithm, which uses the localization precisions of SMLM data to extract surfaces from them (Marin et al., 2023), and the surfaces’ mean curvatures were calculated (Taubin, 1995) as implemented in PYMEVisualize (Marin et al., 2021). To determine the Rtn4 density near certain surface curvatures, the maximal principal curvatures along the surface were divided into 50 bins. The Rtn4 density was calculated as the number of Rtn4 localizations within 50 nm of a surface vertex (see Fig. S2 for vertex description and visual) and binned according to that vertex’s maximal principal curvature. “Rtn4 density/Frequency” was calculated by dividing the bin counts for Rtn4 density by the bin counts for the maximal principal curvature. Images were created using PYMEVisualize (Marin et al., 2021) and custom Python scripts.

#### φ Calculation and simulated random data

The φ angles of Rtn4, Sec61β, and KDEL localizations in tubule cross-sections were calculated using a custom Python script. In PYMEVisualize, small ROIs of ER tubules were created from larger datasets. The 3D surfaces for each ROI were generated based on the localizations from the nanobody labeling in each sample and the skeletons were generated from those 3D surfaces. Vectors pointing from the skeleton of the tubule to each localization from the secondary antibody labeling in each sample were calculated. The angles between these vectors and the z-axis (vector normal to the coverslip) were calculated using Eq. 1. We used the skeletons from each dataset to simulate data randomly distributed around them in circular rings to show what the φ distribution would be for such data (Fig. S2 F). The distributions of angles for simulated, Rtn4, Sec61β, and KDEL points were tested for significant differences by calculating the Wilcoxon rank-sum statistic.

#### Single-molecule counting analysis

Single-molecule counting datasets using fluorogenic DNA PAINT probes were localized in PYME. For each dataset, separate ROIs of Nup96 and Rtn4 were created. The Nup96 ROI was fed into super-resolution microscopy platform (SMAP; Thevathasan et al., 2019; Ries, 2020). Circular ROIs of individual nuclear pore complexes (NPC) were first automatically segmented in SMAP followed by manually removing out-of-focus ROIs. Built-in tools in SMAP were used to determine the effective labeling efficiency and the number of localizations per NPC. This information enables the calculation of the number of localizations per Nup96 protein as well as the number of localizations per labeled Nup96 protein. The Rtn4 ROI was segmented with DBSCAN using a search radius of 15 nm and a minimum cluster size of 1 to identify unique clusters of Rtn4 localizations. Small clusters were later filtered out if they contained less localizations than the determined number of localizations per labeled Nup96 for that dataset to ensure the clusters of points contained at least one protein. The number of Rtn4 proteins per cluster was calculated by dividing the number of Rtn4 localizations in each cluster by the number of localizations per Nup96.

#### ψ Calculation

DBSCAN was used to segment 3D point clouds of Rtn4 localizations into unique clusters using a search radius of 12 nm and a minimum clump size of 5. Clusters were analyzed using the “MeasureClusters3D” module within PYMEVisualize (Marin et al., 2021). Specifically, principal component analysis was used to determine the principal axis of the clusters with the highest eigenvalue, which we refer to as the major axis. Vectors ($\vec{N}$) pointing from each cluster’s center to the closest point on the skeleton were calculated. Each cluster’s major axis was projected onto a plane orthogonal to its $\vec{N}$. The ψ angles were calculated by using Eq. 1 with the projected major axes of the clusters and vectors orthogonal to the tubule axes and tangent to the projected plane as input vectors. Only clusters containing >10 localizations and <50 localizations were used for this analysis. This removed background clusters and clusters that were too close together to segment, respectively. The relative distribution of different-sized clusters is shown in Fig. S5 A.

#### Rtn4 3D cluster anisotropy analysis and simulated linear and circular clusters

Circular and linear clusters were simulated to compare to observed Rtn4 clusters. The simulated clusters possessed the same center points, radii of gyration (Rg), and localization counts as the observed Rtn4 clusters. To simulate circular clusters, each point in the cluster was assigned a uniform random angle from 0 to 2π, and the uniform random radius (distance from the cluster center) varied as $\sqrt{x}*Rg$, where x ranged from 0 to 1 and Rg was copied from the observed cluster each individual simulated cluster was based on. To simulate linear clusters, each point in the cluster was assigned the same angle, initially pulled from a uniform random distribution from 0 to 2π, and a uniform random radius from 0 to the radius of gyration. All points in both types of simulated clusters had some uncertainty incorporated into their positions. This was done by adding random numbers from a Gaussian distribution (with μ = 0 and σ = 5.26; the average localization precision of the real data) to each component of their coordinates. The anisotropies of the simulated clusters and the observed Rtn4 clusters were calculated and compared. Clusters were filtered as 10 < localizations < 50 to avoid background clusters and clusters that were too close together to segment.

$$angle=\cos^{-1}\left(\frac{xy}{\left|x\right|\left|y\right|}\right)\text{,}$$ (1)

where x and y are vectors and |x| and |y| are their magnitudes.

The Rg calculated from the observed clusters and used in the simulations are likely overestimates since they are effectively the true Rg convolved by the localization precision. Thus, our linear-shaped simulated clusters will appear slightly more anisotropic than they would if the true Rg was used. Indeed, if we multiply the calculated Rg by a factor of 0.925, the anisotropy distributions for the linear-shaped simulated data shift left and more closely align with the observed cluster data (Fig. S6 E).

#### Simulating clusters with explicit intermonomer distances

The frequency of different oligomer sizes was taken from the DNA-PAINT counting data (Fig. 4). We determined a factor (3.23) that could transform the distribution of localizations per cluster of the 3D data to one that looked identical to that of the distribution of proteins per cluster from the counting data. This factor is roughly how many localizations we identify per protein. This was used as the λ for a Poisson distribution that was drawn from to determine the number of localizations per monomer in our simulated clusters. We added some localization precision to each simulated localization by adding some uncertainty drawn from a Gaussian distribution with μ = 0 nm and σ = 5.26 nm, which is the average localization precision of our real data. The intermonomer distances tested were based on the theoretical calculation of Rtn4b molecules possessing a diameter of ∼4.5 nm, assuming they are roughly spherical. The following website was used to calculate the volume of Rtn4b based on its sequence: http://biotools.nubic.northwestern.edu/proteincalc.html.

#### Estimating the percent area that Rtn4 proteins occupy in the ER membrane

We assume that each Rtn4b protein has a diameter of ∼4.5 nm based on the calculation in the section above. We calculated the area that each Rtn4b protein would occupy in the membrane as π * r^2^. We used the same factor of 3.23 (described above) to roughly convert our 3D localizations of Rtn4 to the number of Rtn4 proteins. We then calculated the percent area that Rtn4 proteins occupy in the membrane using Eq. 2:

$$\frac{\left(\#\;of\;Rtn4\;proteins\right)*\left(Area\;of\;Rtn4\;protein\right)}{Surface\;area\;of\;3D\;surface}\;*\;100\text{.}$$ (2)

#### Statistics

All statistical tests were performed using the SciPy (Virtanen et al., 2020) library of Python functions.

### Online supplemental material

Fig. S1 shows the Western blots of the U-2 OS Rtn4-Halo and U-2 OS Rtn4-mEGFP CRISPR cell lines. Fig. S2 shows examples of point cloud surface fitting, surface vertices, skeletons, and data simulated randomly in a circle around skeletons. Fig. S3 contains nanobody controls for φ angle analyses and a plot comparing the three distributions of Rtn4, Sec61β, and KDEL. Fig. S4 compares the localization distributions of Rtn4 and Sec61β. Fig. S5 shows the orientation preference of Rtn4 clusters of varying size cutoffs and a 2D histogram of φ vs. ψ. Fig. S6 contains the anisotropy distributions of different oligomer simulations. Video 1 shows an overview and scan of the data shown in Fig. 2. Videos 2, 3, 4, and 5 show a tubule with the observed oligomers or the various simulated oligomers on it.

## Supplementary Material

SourceData FS1 is the source file for Fig. S1.

## Data Availability

All data (excluding raw SMLM data) are available at: https://doi.org/10.5281/zenodo.7569008. Raw SMLM data is available from the corresponding author upon reasonable request. Python Microscopy Environment (PYME) is available at https://python-microscopy.org/. All custom scripts were created in jupyter-lab and are available at the GitHub repository: (https://github.com/lukasfue/Rtn4_paper_code).
