# Supplementary material for: The nanoscale organization of reticulon 4 shapes local endoplasmic reticulum structure in situ
Source: J Cell Biol. 2023 Jul 26;222(10):e202301112. doi: 10.1083/jcb.202301112 (PMC10373298; doi:10.1083/jcb.202301112)
Supplement: SourceData FS1 — is the source file for Fig. S1. [file JCB_202301112_SourceDataFS1.pdf]

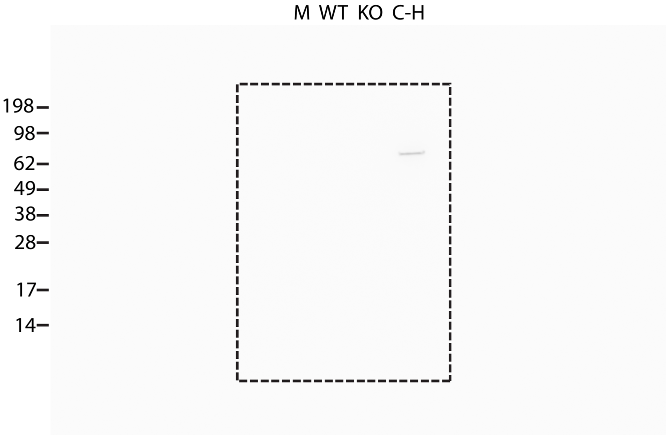

Anti-Halo

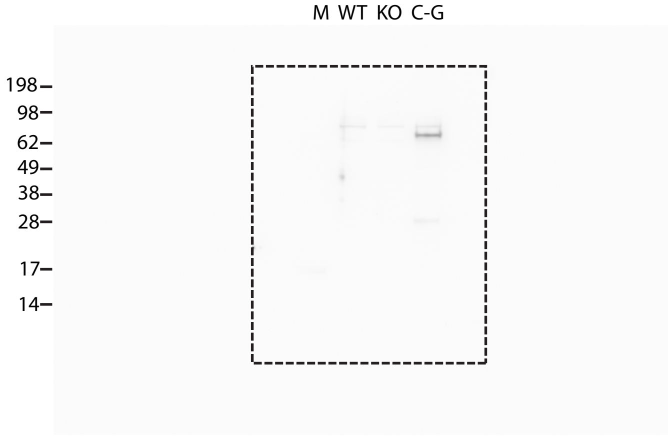

Anti-GFP

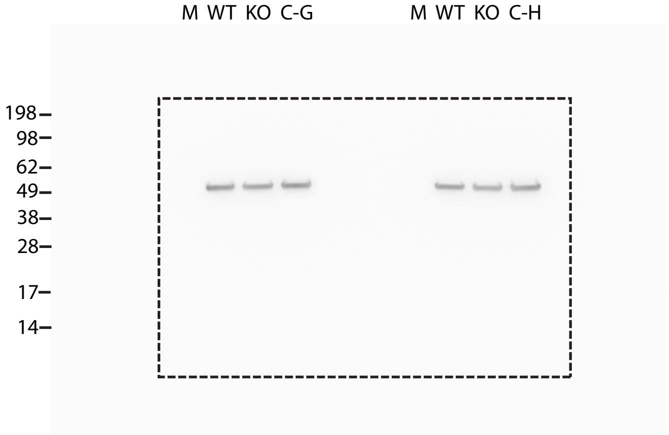

Anti-α-tubulin

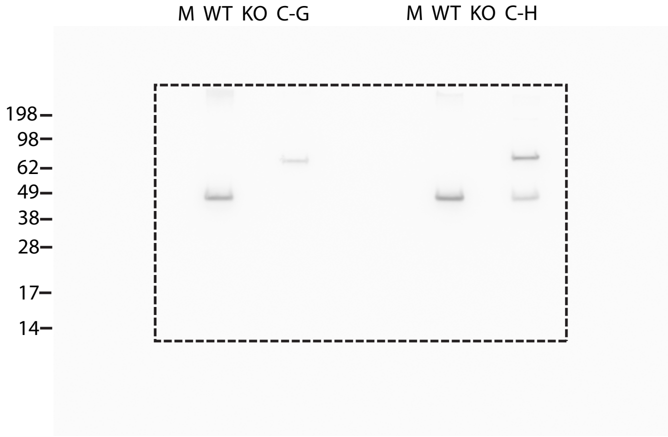

Anti-Rtn4
